# Human-bat contacts in the Netherlands, and potential risks for virus exchange

**DOI:** 10.1186/s42522-024-00132-6

**Published:** 2025-02-15

**Authors:** L. Begeman, M. J. M. Geschiere, W. F. de Boer, J. M. A. van den Brand, P. L. Eblé, J. H. T. C. van der Kerkhof, I. Keur, P. H. C. Lina, C. B. E. M. Reusken, M. de Rosa, M. J. Schillemans, I. Schreuder, C. M. Swaan, K. van Zoonen, T. Kuiken

**Affiliations:** 1 Department of Viroscience, Erasmus University Medical Centre, Rotterdam, The Netherlands; 2 Wildlife Ecology and Conservation Group, Wageningen University, Wageningen, The Netherlands; 3 Division of Pathology, Faculty of Veterinary Medicine, Utrecht University, Utrecht, The Netherlands; 4 Dutch Wildlife Health Centre (DWHC), Utrecht University, Utrecht, The Netherlands; 5 Wageningen Bioveterinary Research, Lelystad, The Netherlands; 6 National Coordination Center for Infectious Disease Control, National Institute for Public Health and the Environment, Bilthoven, The Netherlands; 7 Incident and Crisis Centre, Netherlands Food and Consumer Product Safety Authority, Utrecht, The Netherlands; 8 Naturalis Biodiversity Center, Leiden, The Netherlands; 9 Dutch Mammal Society, Nijmegen, The Netherlands

**Keywords:** Chiroptera, Human-bat interface, Lyssavirus, Questionnaire, Virus, Zoonoses

## Abstract

**Background:**

Contacts between people and free-ranging animals have a potential to cause viral disease epidemics when novel viruses are exchanged. The Netherlands has approximately 18 native bat species, of which some generally use buildings for roosting, and has a dense human population. Frequent indirect and direct contacts between bats and humans could thus be expected, however, this has hardly been studied.

**Methods:**

To study human-bat contacts, people living in the Netherlands were questioned about the type and frequency of their bat contacts, their bat knowledge and perception of bats. For analyses respondents were grouped into (1) general population, (2) bat contact risk group, and (3) people that live in a house with a roost site for a Common Pipistrelle Bat maternity group. Associations between human-bat contacts and other variables were tested by an ordinal logistic regression model.

**Results:**

We show that 85% (226/265) of group 1 reported no contacts, while 11% (28/265) reported indirect, and 4% (11/265) direct contacts with live bats, dead bats or bat products as their closest type of contacts. These contacts occurred mostly less than yearly. Somewhat similarly, the majority, 69% (9/13) of group 3 reported no contacts, and 15% (2/13) reported indirect contacts and 15% (2/13) reported direct contacts. These occurred monthly to less than yearly. In contrast, a minority, 5% (11/227) in group 2 reported no contacts, while 37% (85/227) reported direct bat contacts, mostly yearly, and 38% (86/227) reported bat-related injury, mostly less than yearly, as their closest type of contact. Overall, an increase in knowledge on bats and bat-related diseases was correlated with closer bat contacts.

**Conclusions:**

We conclude that even though bats live close to people in the Netherlands, direct contacts between bats, or bat products, and humans are rare in people from the general population, while being common in people involved in bat-related work. Mitigation of human-bat contacts will be most efficient when targeted to specific groups that are likely to have contacts with bats.

## Background

Viral zoonotic diseases can be devastating for individuals as well as for society. Due to several factors, including changes in human-animal contacts, there is a concern that (viral) zoonotic diseases will emerge more frequently in the future [1]. Viral zoonoses can be transmitted from animals to humans via various contact routes and types of interactions. Contacts can be direct, e.g. rabies virus transmission via a bite, or indirect e.g. Nipah virus transmission via contaminated food [2]. Zoonotic viruses can also transmit indirectly from a natural reservoir species, via an intermediate host, to humans as in the case of Hendra virus, which is spread from Pteropodid bats to humans via infected horses [3]. The virus’ ability to infect via a certain contact depends on both virus and host characteristics, as well as the type of contact that takes place. Therefore, to learn how to efficiently prevent the emergence and transmission of zoonotic diseases, it is important to learn not only about virus and host characteristics, but also about human-animal contacts and behaviour leading to those contacts.

Understanding these human-animal contacts is also important for infectious disease transmissions from humans to animals, as human diseases can be transmitted (back) to animals, reverse zoonosis or anthropozoonoses [4]. When a reverse zoonosis occurs, it is not the humans, but the animals involved that might suffer morbidity and mortality from the disease. In addition, the animal population might be impacted by human mitigation measures like expulsion or culling [5, 6], to prevent transmissions back to humans again. As global biodiversity loss is a big concern of our time [7] it is also relevant to prevent reverse zoonoses.

Bats comprise approximately one fifth of all mammal species. This makes bats a potential source of a great variety of viruses, of which some can be (potentially) zoonotic [8]. Eighteen different bat species regularly occur in the Netherlands. Some of these live frequently close to people, for example because they use buildings to roost. This lifestyle [9] can be expected to lead to contacts, direct or indirect, with humans and domestic animals like cats. At least one zoonotic virus is circulating in a bat species that occurs in the Netherlands: this is European bat lyssavirus type 1 which circulates in Serotine Bats (*Eptesicus serotinus*) [10]. The virus infection can cause the fatal neurologic disease rabies in both bats and humans. Previous studies reported on bat-human contacts for wildlife rehabilitators specifically [11] or reported on human contacts occurring with rabid bats specifically (Netherlands, France) [12, 13]. The human-bat contacts for a much larger, more general public in the Western European geographical and cultural setting remains unknown.

The aim of our study was to investigate types and frequencies of human-bat contacts in the Netherlands, to learn more about its potential for virus transmissions between people and bats. The influence of human variables, like age, gender, education and bat-related knowledge and perspectives, on the occurrence of human-bat contacts were additionally evaluated. We studied human-bat contacts via questionnaires for three different groups: (1) General population in the Netherlands; (2) ‘Bat contact risk’ group including e.g. bat rehabilitators and people monitoring bat roosts (3) People who live in buildings where Common Pipistrelle Bat (*Pipistrellus pipistrellus*) maternity groups are dwelling as these are by far the most common and widespread bat species occurring in the Netherlands, mostly dwelling in buildings.

## Materials and methods

### Questionnaire

We studied type and frequency of human-bat contacts via a hard copy or a digital questionnaire. Participants were specifically asked to answer the questions about their bat contacts regarding situations that had occurred in the (continental) Netherlands, and only about the period of five years preceding the moment of filling out the questionnaire. To learn how human-bat contacts relate to the respondent’s potential motivation for seeking or avoiding bat contacts, the questionnaire included questions regarding how people perceived or valued bats, and regarding their knowledge about bats, as well as about bat rabies. A part of the study respondents was also asked to donate blood, so that answers to the questions could be related to the detection of antibodies against bat viruses. The antibody detection data will be reported separately. The questionnaire consisted of four parts (List 1). Respondents’s answers only were used if all the questions in the questionnaire had been answered.


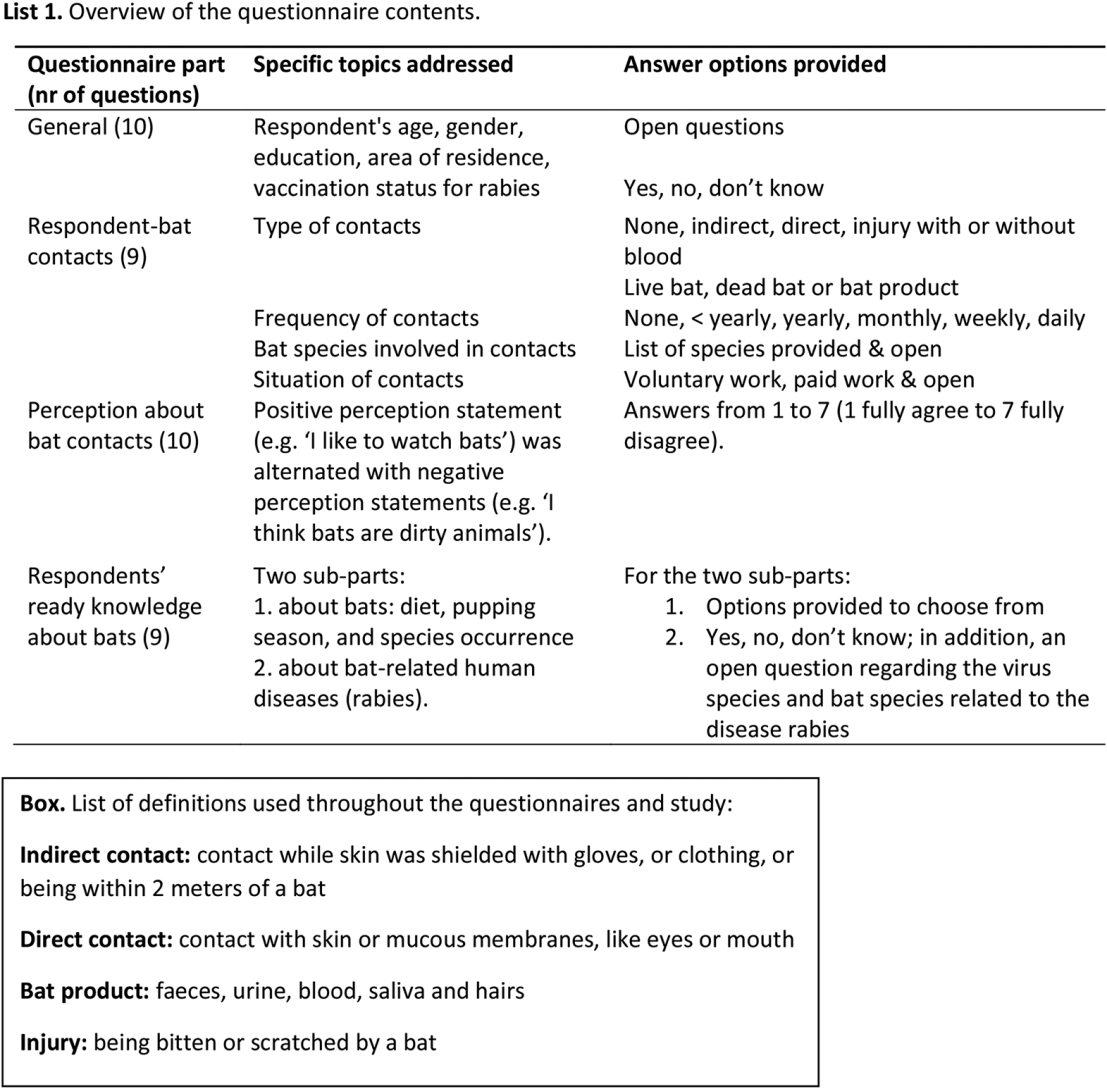


### Respondents’ inclusion into three groups

1. General population in the Netherlands: To get a representative cross section, the questionnaire was sent out to a panel of a commercial research organization (Flycatcher, www.flycatcherpanel.nl). The questionnaire was digitized and sent out to 500 people 18 years or above and matched with regards to their gender, age, education and province of residence to match the Dutch population. Participants were recruited and included at the end of 2020, during the COVID-19 pandemic. The number of people who responded to the questionnaire was 314 (62.8%) which is an ’average’ response rate (between 60 and 65% is seen as standard for the Flycatcher panel). From this group, 14 people were excluded as they did not complete the questionnaire for unknown reasons, 8 were excluded as they indicated the bat contact occurred in a zoo or outside the Netherlands, and another 27 people were excluded because they indicated they had bat contacts because of their (voluntary) jobs. As such, the total final number of included respondents in this group was 265.
2. Bat contact risk group: respondents, 18 years old or above, were recruited at various bat-related events. These events occurred between 2018 and 2019 (all before the COVID-19 pandemic). Questionnaires were handed out in paper format. Recruitment took place twice at the event of a VLEN meeting (Vleermuiswerkgroep Nederland, Bat working group of the Netherlands, Dutch Mammal Society) (148 respondents), once each at events for bat rehabilitators (*n* = 15), city ecologists (Vleermuizen in de stad) (*n* = 14), and animal rescue and shelter people (*n* = 23). Via the Flycatcher panel described above, another 27 respondents were included in this group, because they indicated to be active in (voluntary) jobs that are more likely to be associated with contacts with bats. As such, the total number of respondents in this group was 227. Between group 1 and group 2, there were no significant differences in age (Mann-Whitney U Test, *p* = 0.076) and gender (Chi-Square Test, *p* = 0.496). Group 2 had a slightly higher education level than group 1 (Chi-Square Test, *p* < 0.001).
3. Residents of buildings that are also used by Common Pipistrelle Bat maternity groups: From spring to end of the summer, female Common Pipistrelle Bats roost together in groups, ranging in size from 20 to 100 bats. Common Pipistrelle Bat maternity colonies are monitored by volunteers (Bat 030, bat volunteer group municipality Utrecht), in a city in the centre of the Netherlands. Volunteers explained to human residents about bats, about our project, and asked residents for permission to keep watch near their building to observe and count bats flying in or out, as well as to put down sheets on or near their property for sampling bat faeces. The second subsequent year (end of 2020, during the COVID-19 pandemic) the same 35 buildings were visited by volunteers. This time volunteers explained about the project and asked resident(s) to fill out a paper version of the questionnaire to learn about their bat contacts. An envelope was included to make it easy, and without extra costs, to send the questionnaire back to the research institute. Residents of 4 of 35 (11%) buildings were excluded from the study because they could not read Dutch. Residents of another 5 of 35 (14%) buildings were excluded because they were not at home. Lastly, residents of another 3 of 35 (9%) buildings were excluded because they refused the invitation. These residents indicated lack of time, lack of motivation, or never filling out questionnaires, as reasons for refusal. Of the 23 of 35 residents that accepted the questionnaire, 13 (57%) returned the questionnaire to the researchers. All 13 questionnaires had been completed and as such, the total number of respondents in this group was 13.

To prevent a person being included in our study more than once, we asked prior to inclusion to retract from the study if they had participated in our study before. Recruitment for these groups is outlined with more detail below.

### Analyses and statistics

For analysis of the respondents’ perception questions, each of the 10 answers was scored by use of a Likert scale, from 1 (fully agree) to 7 (fully disagree) and totals were added. The scores for the negative perception statements were reversed (so that 1 becomes 7, 2 becomes 6 etc.) so each respondent received an overall score between 10 and 70, where higher scores related to respondents that perceived bats more positively.

For analyses of the respondents’ ready knowledge, two parts were assessed separately: knowledge about bats, and bat-related diseases. Respondents received one point for each correct answer. In this way, bat knowledge part scores were possible between 0 and 15. For the diseases part scores were possible between 0 and 7. For each part, higher scores related to respondents with a better knowledge.

To describe the respondent-bat contacts percentages were used. To understand if, and how much, some selected variables were correlated with human-bat contacts, we tested for associations between human-bat contacts and other variables from the questionnaire by using an ordinal logistic regression model. In this model we reclassified the dependent ordinal variable, *the bat contact level*, to four categories, for all respondents. As before we classified based on, using the respondent’s closest reported bat contact: (1) **No** indirect or direct bat contacts (2) **Indirect** contact with a living bat, bat product or dead bat (3) **Direct** contact with a living bat, bat product or dead bat without injury (4) Direct contact with a bat that caused **injury** with or without blood. We used continuous and nominal variables in the model. Continuous covariates were age, perception score (scores 10–70), bat knowledge score (0–15), zoonotic bat virus knowledge score (0–7). Nominal variables were education level (8 groups from no education to academic), gender, and inclusion group (three: general public, bat contact risk group, and residents of houses with roosts). We reduced the full model using a backward elimination of variables that were not significant, i.e. using *p* < 0.05 as threshold.

### Ethics

This questionnaire was part of the Zoonoses in the night project, which was evaluated and approved by the Medical Ethical Committee of Erasmus Medical Centre (MEC-2018-102; NL64612.078. 18, v4).

## Results

### Bat contacts reported for general population group

**Fig. 1:**
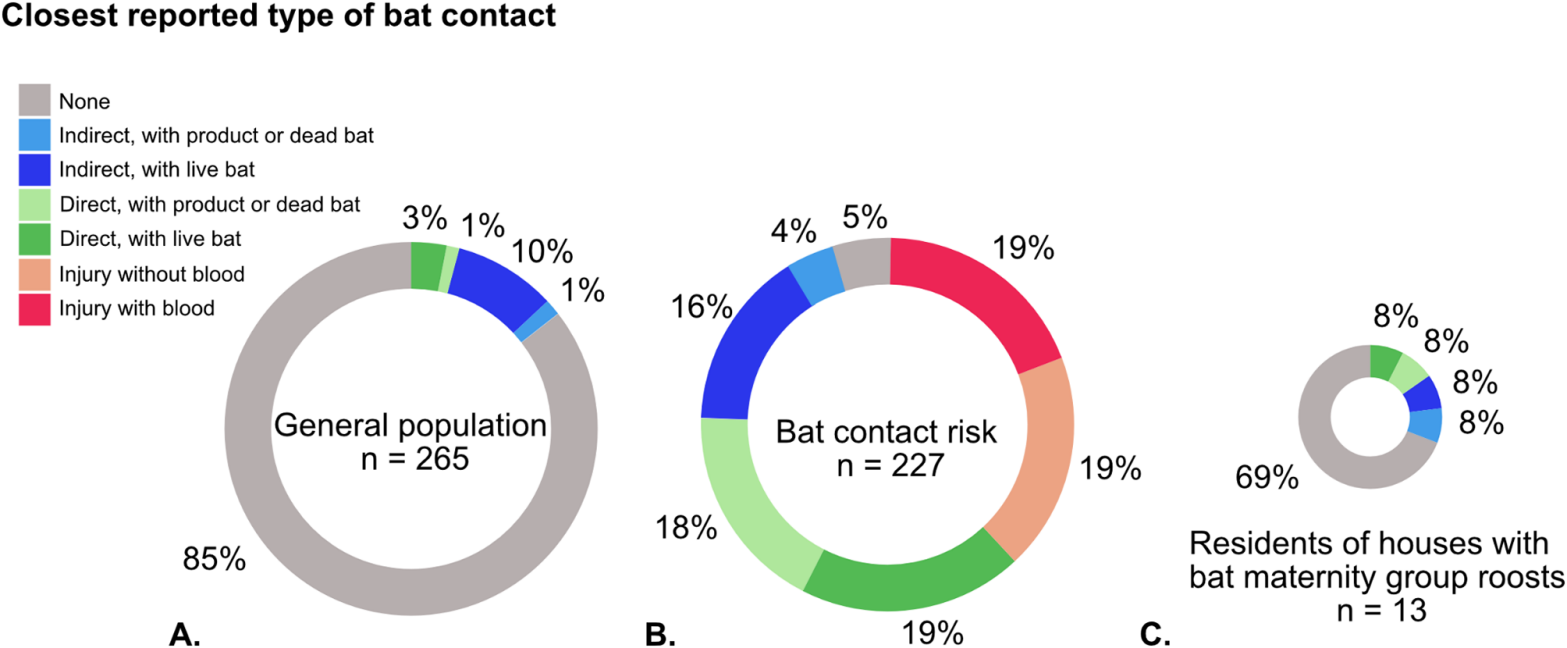
Pie charts showing closest types of bat contact reported by respondents from the general population (**A**), from the bat contact risk group (**B**), and residents of houses with Common Pipistrelle Bat maternity groups (**C**). In the general population (**A**) most respondents did not report any contacts, while in the bat contact risk group (**B**) three quarters reported direct bat contacts or injury from bats as their closest contact

**Fig. 2:**
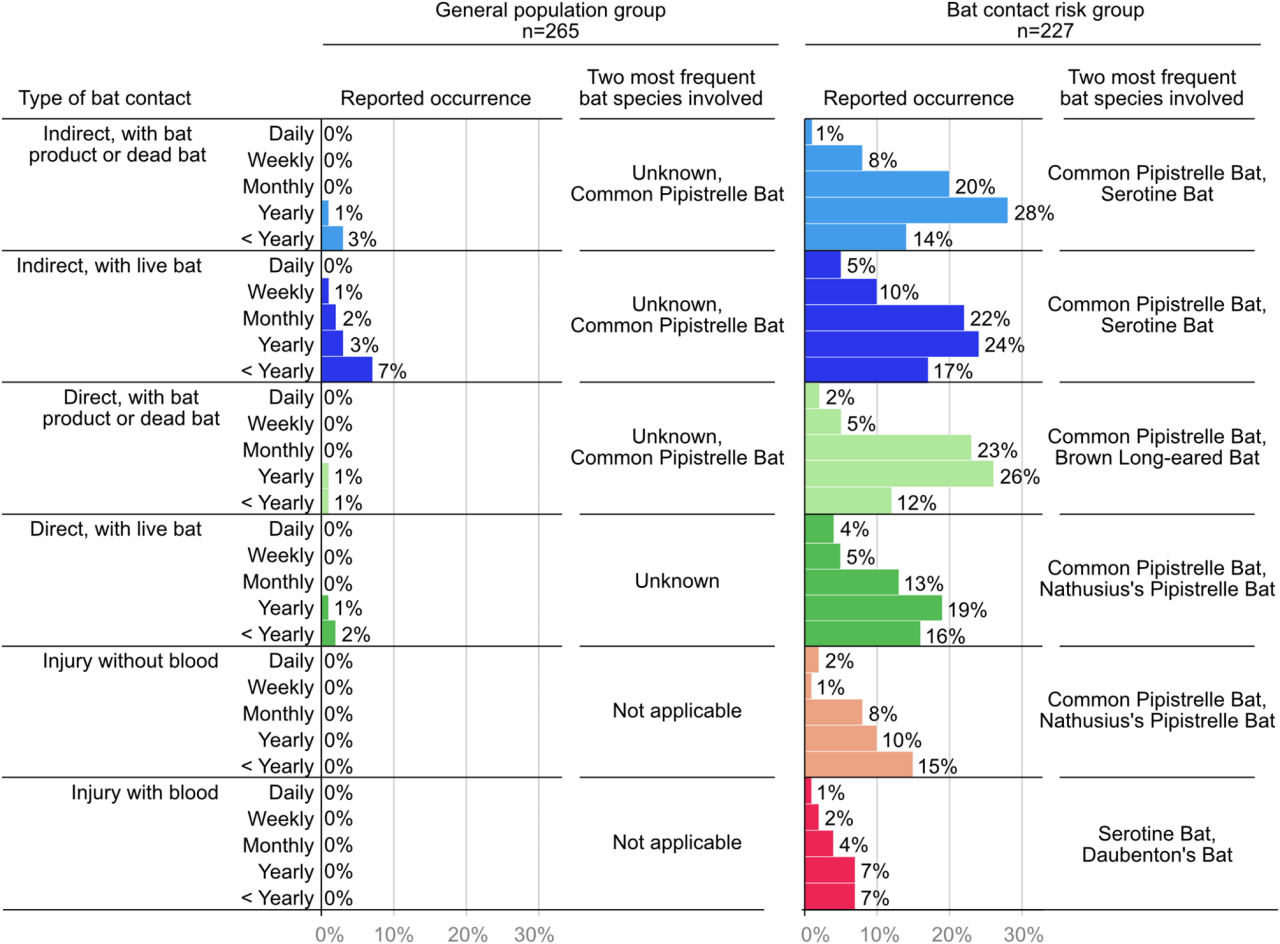
Frequencies of bat contacts per contact type. Percentages of respondents who reported a certain frequency (daily to less than yearly) per contact type, as well as the two most commonly involved bat species. Overall, Common Pipistrelle Bats were the most frequently involved bat species. Probably due to their small teeth and biting force pipistrelles are less likely to cause injury with blood

In our general population group, direct and indirect contacts with a live bat, dead bat, or bat products, were reported by a small proportion of participants (Fig. 1A), and if reported the overall frequency was mostly yearly, or less than yearly (Fig. 2). The biggest part (85%, 226/265) reported to have had no direct or indirect contacts with bats or bat products in the Netherlands at all in the past five years. Most contacts occurred in one of the indirect contact types with a live bat, dead bat or bat product. The respondents who reported *any* bat contacts (indirect, direct, with dead bats, live bats or bat products) (39/265) indicated contact(s) had occurred in their garden (14/39), ‘at home’ (11/39), outside (7/39), or in/at a building they visited (3/39). For the people reporting contact(s) to occur ‘at home’ it was not clear if this was *near*, or *in* their home.

A small part (4%, 11/265) of the respondents from the general population reported direct contact with a live bat, dead bat or bat product as their closest contact. These combined direct contacts were reported mostly to occur yearly or less (91%, 10/11). The exception was one respondent who reported monthly direct contact with a live bat. This contact was stated to occur in the respondent’s barn, which was inhabited by bats. The respondent did not know which bat species it was. None of the 265 respondents reported they had an injury caused by a bat.

Respondents in the general population group who indicated any contact with a live bat, dead bat or bat product (39/265, 15%) usually did not know the bat species with which the contact had occurred. In seven reported bat contacts the bat species was indicated to have been a Common Pipistrelle Bat and the type of contact was indirect contact with a live bat, dead bat, or bat products (*n* = 6) or direct contact with a dead bat or bat products (*n* = 1). Common Pipistrelle Bats are the most common and widespread bat species in the Netherlands, and they mostly roosts in buildings [14]. Yearly direct contact with a bat product or dead bat, and indirect contact with a live bat were also reported with a Leisler’s Bat (*Nyctalus leisleri*) by one respondent. Further details from the questionnaire indicated this contact occurred at home. This is remarkable as this bat species is not very common in the Netherlands, and uses trees rather than buildings for roosting [15]. There might be a misunderstanding related to the Dutch name for this species which is ‘forest bat’, which might have been misunderstood by the respondent as being a bat from the forest. This respondent also reported to be a cat owner, and that the cat had brought home a bat or bats. As no other bat contacts were reported by this respondent, this supposed contact with Leisler’s Bats likely originated from the cat bringing home these bats.

### Bat contacts reported for bat contact risk group

Reported closest types of bat contacts in the bat contact risk group were much different from the general population group (Fig. 1B). Indirect contact with a bat or bat product as their closest contact to bats was reported by a minority of respondents, 20% (45/227), while approximately equal proportions reported direct contacts (37%, 85/227) or injuries (38%, 86/227) as their closest types of contacts. Overall, indirect contacts with a live bat, dead bat, or bat products were reported most frequently, 340 times (Fig. 2) and were reported to occur mostly yearly or less (56%, 190/340). The majority of these contacts (89%, 40/45) occurred during voluntary (*n* = 25) or paid (*n* = 15) work.

Direct contacts with live bat, dead bat, or bat products were reported 280 times (Fig. 2) and were reported to occur mostly yearly or less (59%, 165/280). These contacts occurred during voluntary (*n* = 43) or paid (*n* = 26) work, or both (*n* = 16). An additional situation reported by two respondents was when their cat had brought home a bat.

Bat contact that resulted in injury from a bat was reported 130 times, of which about one third resulted in bleeding (36%, 47/130). The frequency of injury (with or without blood) contact type was reported to occur mostly yearly or less (68%, 88/130) (Fig. 2). These contacts occurred during voluntary (*n* = 59) or paid work (*n* = 7) or both (*n* = 20), but educative courses (like a course to learn how to catch bats in nets) were mentioned as an additional situation in which the contact had occurred.

In the bat contact risk group, those that reported any type of contact (95%, 216/227), the bat species with which the contact had occurred was usually reported. Eighteen species of bats occur regularly in the Netherlands, and contacts were reported with almost all of these, but some were reported more frequently than others (Fig. 2). Per contact type, the relative frequency of bat species was very similar, aside from injury with blood. For injury with blood, Common Pipistrelle Bats, Nathusius’s Pipistrelle Bats *(Pipistrellus nathusii)* and Brown Long-eared Bats (*Plecotus auritus*) were mentioned relatively less frequently compared to the other types of contact, while Serotine Bats, Daubenton’s Bats (*Myotis daubentonii*), Pond Bats (*Myotis dasycneme*) and Noctule Bats (*Nyctalus noctula*) were mentioned relatively more frequently. Overall, species had the following order of highest to lowest number of being mentioned in the questionnaire, combined for all types and frequencies of contacts, so the number is not corrected for the reported frequency of the indicated contact (daily, weekly, monthly, yearly, less than yearly): Common Pipistrelle Bat (*n* = 554), Nathusius’s Pipistrelle Bat (*n* = 336), Serotine Bat (*n* = 325), Brown Long-eared Bat (*n* = 300), Daubenton’s Bat (*n* = 228), Noctule Bat (*n* = 186), Pond Bat (*n* = 177), Natterer’s Bat (*Myotis nattereri*) (*n* = 133), Whiskered Bat (*Myotis mystacinus*) (*n* = 133), Greater Mouse-eared Bat (*Myotis myotis*) (*n* = 85), Parti-coloured Bat (*Vespertilio murinus*) (*n* = 84), and Leisler’s Bat (*n* = 76). Very rarely reported (one to four) were contacts with Geoffrey’s Bat (*Myotis emarginatus*), Brandt’s Bat (*Myotis brandtii*), Bechstein’s Bat (*Myotis bechsteinii*), Soprano Pipistrelle Bat (*Pipistrellus pygmaeus*), and Grey Long-eared Bat (*Plecotus austriacus*). No contacts were reported for the Western Barbastelle Bat (*Barbastella barbastellus*).

### Bat contacts reported by people that live in a house containing a roost side for Common Pipistrelle Bat maternity groups

The majority (69%, 9/13) indicated to have had no contacts with live bats, dead bats, or bat products, while 1 of 13 (8%) respondents indicated to be in each of the indirect and direct contact types (Fig. 1). None reported injuries. Only the person that reported direct contact (less than yearly) with a live bat knew the species, a Common Pipistrelle Bat. Other bat contacts occurred monthly (indirect contact bat product *n* = 1, indirect contact with a live bat *n* = 1, direct contact with bat product *n* = 1) or yearly (indirect contact bat product *n* = 1). The circumstances in which these contacts had occurred were all situations occurring at home. The direct contact with a live bat was due to finding a stranded bat that originated from the cavity wall. The direct contact with a bat product was direct contact with bat faeces as an effect of cleaning terrace furniture. The indirect contacts were from being close to a bat hanging near a window, or because of sweeping faeces from the pavement.

### Bat-cat contacts

Taking information from all respondents together, groups 1, 2 and 3, 34% (173/505) indicated to have a cat, or cats as a pet. Of these, 5% (8/173) observed their cat or cats catching a bat. To determine whether bats might avoid roosting in buildings where cats were resident, we compared if respondents with known presence of Common Pipistrelle Bat maternity groups in their houses less commonly owned cats, compared to all other respondents for which was assumed the majority would not have a Common Pipistrelle Bat maternity group in their houses. There was no correlation. Five of thirteen respondents (38%) living in houses with bat maternity colonies (group 3) reported to have one or more cats, which was not significantly different from the other respondents (34% [168/492], X2 [2, *n* = 505] = 0.1047, *p* = 0.75).

### Associations between human-bat contacts and human variables that might influence those contacts

For these ordinal logistic regression analyses four ordinal levels of closest reported bat contacts were used as explained in the methodology, in short the four categories were: (1) **No** bat contacts (2) **Indirect** bat contact (3) **Direct** bat contact without injury (4) Direct bat contact with **injury**. Age, gender, and education were not significantly correlated with the reported closest bat contact, and therefore removed from the final model (Table 1).

**Table 1:** Output of the ordinal logistic regression (coefficient, standard error, *t*- and *p*-value) for each of the selected variables in the final model using backward elimination, namely the continuous variables related to knowledge on bats, on bat-related diseases, and bat perception score, and the nominal variable inclusion group: either people from the general public, living in a house with a bat maternity colony, or working in the bat contact risk group. The bat contact risk group was the reference category. Total number of participants *n* = 505

Variable	Coefficient	Standard error	t value	*p* value	Odds ratio	Confidence intervals
Bat disease knowledge	0.202	0.070	2.904	0.004	1.224	1.069–1.405
Bat knowledge	0.120	0.039	3.05	0.002	1.127	1.045–1.219
Bat perception	0.032	0.010	3.277	0.001	1.033	1.013–1.053
Inclusion group						
General public	-3.767	0.300	-12.528	< 0.001	0.023	0.013–0.041
House with bats	-2.808	0.661	-4.25	< 0.001	0.060	0.015–0.207
**Intercepts**						
None - Indirect bat contacts	0.525	0.584	0.900	0.368		
Indirect - direct bat contacts	2.201	0.574	3.832	< 0.001		
Direct bat contacts - injury from a bat	4.090	0.608	6.723	< 0.001		

Positive bat perception scores, higher bat knowledge, and higher bat disease knowledge scores significantly increased the probability of a respondent to be in the two closer types of bat contact categories (i.e. direct contacts without injury, and direct contacts with injury). Another significant predictor for being in a closer type of bat contact category (e.g. direct contacts with or without injury) was if a participant was part of the bat contact risk group, while participants of the general population group and participants that had bat maternity colonies in their houses had a lower probability of falling in this closer type of bat contact category. The two lowest bat contact categories (i.e. none-indirect) could not be statistically distinguished by the model, while the highest two categories (i.e. direct, and direct with injuries) were statistically different from the indirect contact category.

### Lyssavirus-transmission-specific risks

**Fig. 3:**
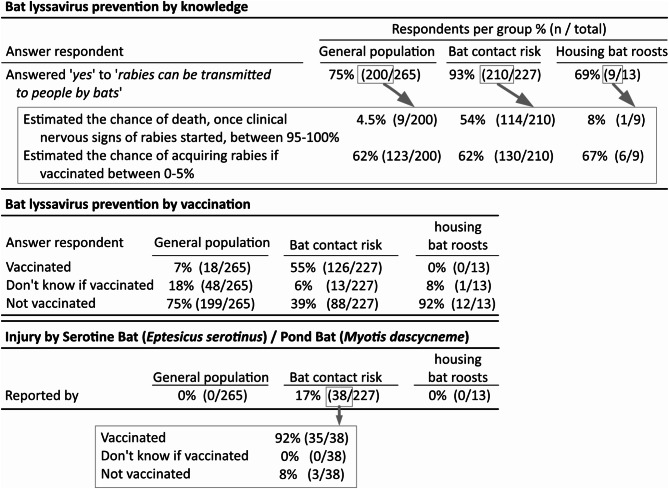
Questionnaire results related to rabies prevention in three risk groups. Serotine Bats and Pond Bats are the two species in the Netherlands in which lyssaviruses have been detected. Data show severity of disease is widely underestimated, as is prevention by vaccination. Not all people that had injury from two bat species that might transmit lyssaviruses were vaccinated

We evaluated the questionnaire results related to risks of transmission of lyssaviruses occurring in bats in the Netherlands. European bat lyssavirus type-1 circulates in Serotine Bats and European bat lyssavirus type− 2 in Pond Bats. We evaluated respondent’s knowledge that helps to prevent lyssavirus infection, vaccination status, and reported injuries with Serotine or Pond Bats (Fig. 3).

Overall, most respondents from all three groups showed to be aware that rabies can be transmitted from bats to people, but gravely underestimate the chance of dying from the disease. However, many more respondents from the bat contact risk group estimated the chance accurately than from the other two groups. Slightly over 60% of respondents from all three groups showed to be aware that vaccination prevents the disease.

Based on rabies risk related injury *not* being reported by 265 respondents of the general population group, we can roughly estimate an upper limit for its prevalence. For this we assume that our respondents are representative for the part of the Dutch population that is not a bat worker, and that respondents filled out the questionnaire correctly. By using the ‘rule of three’ [16] we can be 95% certain that in the general Dutch population the prevalence of rabies risk related injury is at least lower than about 1% (3/265) per five years (the period for which respondents were asked to report bat contacts).

Three unvaccinated people, all in the bat contact risk group, reported Serotine or Pond Bat-related injury, indicating a lyssavirus transmission risk had occurred. Two respondents indicated the injury occurred less than yearly (in the five year period questioned), and one indicated the injury occurred yearly. Trying to estimate a risk related injury in this group per year from these data, means a minimum prevalence of about, 0.4% (1/227, CI: 0.01–2.4%) per year, and a maximum prevalence maximum of 0.6% (1.4 [namely 1 + 0.2 (1/5) + 0.2 (1/5)] /227; CI: 0.1–3.1%) per year. Further zooming in into the rabies knowledge of these three unvaccinated respondents, all three indicated knowing that bats can transmit rabies to people. Their estimations for dying, once they would acquire rabies (defined as neurologic disease due to lyssavirus infection) were 40%, 80% and 95% respectively, while their estimations for dying of rabies if they would have been vaccinated prior to exposure were 0%, 10% and 25% respectively. Thus, knowledge regarding both the severity of the disease, as well as prevention by vaccination was not optimal in these respondents and might have contributed to this lyssavirus transmission risk.

## Discussion

Our results provide information on the self-reported contacts that occur between people and bats in the Netherlands, which can support analyses regarding risks for virus transmissions between bats and humans. Perhaps not surprisingly, overall, less bat contacts were reported by the general Dutch population and people living in a house containing a bat maternity colony, than by a bat contact risk group which included people visiting bat-related events, as well as people performing bat-related work. The extent of the difference might be somewhat surprising. For example, while only 3% of the general public group reported direct contacts with a live bat, none of which were associated with injury, 57% of the bat risk contact group reported direct contacts with a live bat, and 38% reported bat-related injury. These differences are substantial and should be considered in the development of risk mitigation measures, for example monitoring bat workers after bat bites.

Prevalence of direct contacts with bats have previously been studied in other regions. In USA and Canada, bat contacts were reported by 0.0098% of 36,445 participants, when questioned about the last year [17]. In Australia, bat contacts were reported by 5.1% of 821 participants, when questioned about the last five years [18]. Higher prevalences of direct human-bat contact were reported from studies performed in Nigeria (10%) [19], Ghana (66%) [20] and West Java, Indonesia (45%) [21] in specific communities. Most of the bat contacts in these studies occurred due to specific behaviours that involved close contact and/or close proximity with bats, like bat capture, bat rehabilitation, bat hunting or visiting bat caves. These relatively high percentages of bat contacts amongs respondents fit with our percentages in the bat contact risk group (which included people visiting bat-related events, as well as people performing bat-related work). Our results and those of others [19–24] suggest that factors like specific interest in bats, bat-related work, and cultural incentives to visit bats at their roost sites (like gaining manhood), greatly influence the type and frequency of human-bat contact. The difference in prevalences is so big that identification of risk groups might be key for efficient mitigation strategies, as suggested by others [23, 25].

Human behaviour towards bats is expected to be influenced by both knowledge and risk perception. Our results showed that respondents with a higher knowledge level of bats and bat-related diseases, and perceived less risks from bats, had a higher chance of reporting closer type of contacts with bats. Partly this is counterintuitive as the people that are aware that in rare occasions bats can transmit a deadly disease, also have closer bat contacts. The explanation might well be that this is due to the limited number of people reporting any contacts with bats in the general population group, and a confounding factor for the respondents in the bat contact risk group: people that are expected to have direct contacts with bats are more likely to have been informed about rabies risks. Not only because the same people visit bat-related lectures, but also people having been bitten by a bat, might have an increased chance of studying the risks involved themselves, or being informed by having received medical assistance. Another explanation is ‘familiarity breeds contempt’. When someone is exposed often to a known risk, and this generally has no consequences, it might lead to that person losing the initial respect for that risk. In the Netherlands, people that for their work handle bats, are asked by a regulating committee, to show a recent serologic titre check to avoid rabies infection. Because of this, people handling bats might feel well protected not only for rabies, but in general, and this might make people less careful and increase the chance of being bitten. Because of these reasons our results do not show a straightforward and expected correlation between better knowledge of bat-related diseases and less bat contacts.

Bat lyssaviruses are transmitted to people through bites or scratches inflicted by infected bats of a few bat species. Most people in our general population group (75%), and bat contact risk group (93%) did know that rabies can be transmitted to people by bats, however, subsequent knowledge of the severity and prognosis of this disease was lacking (4.5% general population and 54% bat contact risk group had the question right). In addition, the knowledge on preventability by vaccination (~ 62%) was not optimal. Our findings suggest that most people from the general population might not be motivated, based on risk on rabies alone, to seek contact with health care professionals after acquiring a bat-related injury. This lack of knowledge identified here is of concern for rabies prevention. Based on our results we estimated the prevalence of bat-related injury amongst the general public in the Netherlands to be at least *less* than 1% per five years (95% confidence). A previous study from the Netherlands that investigated rabies risks due to bat bites, reported a frequency of 17 bat bites in the Netherlands in a five-year period; assuming a population of 16 million the five-year prevalence is 0.0001% (17/16,000,000) [12]. As Takumi et al. only reported the people that sought medical care, and not everyone having a bat-related injury might seek medical care, the actual incidence might be higher. It would be good to get a closer estimate of the prevalence of bat bites, however a much larger group than our current one should then be questioned about the occurrence of bat-related injury.

Besides the risk of acquiring rabies by a bite of a lyssavirus-infected bat, risks for human-bat contacts in the Netherlands are not well known. For example, other bat lyssaviruses that might be zoonotic have been detected near the Netherlands in bat species that also occur in the Netherlands [26–28], and new bat lyssaviruses are regularly being discovered in bat species that previously were not studied [29, 30]. Other viruses than lyssaviruses have been detected in bats in the Netherlands and bordering countries, but their zoonotic potential is not clear [31–33]. Vice versa, we know very little about the risks of humans transmitting viruses to bats (reverse zoonosis). If such a human-origin virus transmits well between bats, it might have detrimental effects on bats, of which some species are already endangered. Therefore, limiting risky human-bat contacts—without negatively affecting bat conservation and relevant bat research—is beneficial both for human and bat health.

## Conclusions

Clear differences in bat contacts were shown between a group representing the general population and a group identified as having increased risk of bat contacts. Further identification of certain risk groups might be key for efficient mitigation strategies. Overall direct contacts between live bats and members of the public are rare in the Netherlands and therefore the risks for virus exchange between humans and bats seem small.

## Data Availability

The datasets generated and analysed during the current study are available in the DANS narcis repository, 10.17026/dans-xqr-tgza.
